# Efficient Sulfur Host Based on Yolk‐Shell Iron Oxide/Sulfide‐Carbon Nanospindles for Lithium‐Sulfur Batteries

**DOI:** 10.1002/cssc.202002731

**Published:** 2021-02-02

**Authors:** Dongjiu Xie, Shilin Mei, Yaolin Xu, Ting Quan, Eneli Härk, Zdravko Kochovski, Yan Lu

**Affiliations:** ^1^ Department of Electrochemical Energy Storage Helmholtz-Zentrum Berlin für Materialien und Energie Hahn-Meitner-Platz 1 14109 Berlin Germany; ^2^ University of Potsdam Institute of Chemistry 14476 Potsdam Germany

**Keywords:** batteries, electrode materials, lithium sulfides, yolk-shell nanostructures

## Abstract

Numerous nanostructured materials have been reported as efficient sulfur hosts to suppress the problematic “shuttling” of lithium polysulfides (LiPSs) in lithium‐sulfur (Li−S) batteries. However, direct comparison of these materials in their efficiency of suppressing LiPSs shuttling is challenging, owing to the structural and morphological differences between individual materials. This study introduces a simple route to synthesize a series of sulfur host materials with the same yolk‐shell nanospindle morphology but tunable compositions (Fe_3_O_4_, FeS, or FeS_2_), which allows for a systematic investigation into the specific effect of chemical composition on the electrochemical performances of Li−S batteries. Among them, the S/FeS_2_−C electrode exhibits the best performance and delivers an initial capacity of 877.6 mAh g^−1^ at 0.5 C with a retention ratio of 86.7 % after 350 cycles. This approach can also be extended to the optimization of materials for other functionalities and applications.

## Graphical abstract

**The great yolk‐shell spindle**: Fe_3_O_4_−C, FeS−C, and FeS_2_−C are synthesized with a yolk‐shell nanospindle morphology and their electrochemical performance in lithium‐sulfur batteries is systematically studied. The S/FeS_2_−C cathode exhibits better long‐term cycling stability than the S/FeS−C cathode. Moreover, FeS_2_ nanoparticles kinetically facilitate lithium polysulfide (LiPS) conversion reactions.

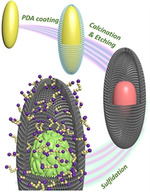

## Introduction

Recently, lithium‐sulfur (Li−S) batteries have attracted a lot of attention due to their high energy density, low cost, and environmental benignity, which is considered to be one of the most promising candidates for the next‐generation battery system.^[1]^ However, the commercialization of Li‐S batteries has been hampered because of the insulating nature of sulfur and Li_2_S, large volume change (ca. 80 %) during charging and discharging, and “shuttle effect” of lithium polysulfides (LiPSs).^[2]^ Currently, tremendous efforts have been devoted to inhibiting the “shuttle effect”, which can be summarized to three approaches according to the diffusion path of LiPSs, including cathode design, separator modification or introduction of interlayer, and electrolyte optimization.^[3]^ Among these approaches, design and synthesis of novel cathode materials with outstanding capability of confinement of the LiPSs on site is substantially efficient and important for the long‐term stability of Li−S batteries. Thus, a lot of efforts have been made to synthesize nanostructures of sulfur host materials. Since the pioneering work by Nazar and co‐workers using the ordered mesoporous carbon (CMK‐13) as the cathode,^[4]^ numerous kinds of porous or hollow carbon materials with hierarchical structures have been designed as sulfur host materials with excellent performance.^[5]^ However, the nonpolarized carbon‐based materials possess a weak binding capability to the polarized LiPSs, resulting in fast capacity decay, especially in long‐term cycling. Instead of weak physical adsorption, many nanostructured and polar metal‐based compounds (hydroxides,^[6]^ oxides_,_ ^[7]^ sulfides,[^1b^, ^8^] nitrides,^[9]^ carbides,^[10]^ and phosphides^[11]^) with strong chemical adsorption of LiPSs were investigated as sulfur host materials for Li−S batteries. However, those metal‐based compounds suffer from low specific surface area and electronic conductivity compared to carbon, leading to a low utilization efficiency of host materials. In addition to introducing metal‐based compounds, covalent C−S bonds can be created in carbon materials by the addition of reactive sulfur‐based intermediates to the unsaturated carbon‐carbon double bonds and the nucleophilic attack of polysulfides with residual oxygen‐containing functional groups in carbon, which could provide chemical confinement of LiPSs.[^9d^, ^12^] More recently, the optimized composites of carbon and metal‐based compounds have been designed by the combination of chemical and physical adsorption of LiPSs. To date, many kinds of nanostructures have been synthesized, including hollow spheres,^[13]^ tubes,^[14]^ cubes,[^7a^, ^9a^, ^11a^] and polyhedra.^[15]^

Besides the nanostructure, the selection of the metal‐based compounds is also crucial for improving the electrochemical performance of Li−S batteries. Therefore, systematical research on the compositions is highly demanded to optimize the chemical compositions of metal‐based compounds. Among various compounds, iron‐based materials have attracted tremendous interest in Li−S batteries because of its nontoxicity, high conductivity and low cost. So far, nanostructure and synthetic methods of the iron‐based compounds have been widely investigated in Li−S batteries.[^7a^, ^16^] For instance, through the polar‐polar interaction between iron oxides and LiPSs, three‐dimensional Fe_2_O_3_‐graphene were designed as the anchor sites for LiPSs. It was found that Fe_2_O_3_ particle can restrain the shuttling of LiPSs and accelerate the transformation of soluble LiPSs into insoluble products.^[7c]^ Besides, yolk‐shell Fe_3_O_4_−carbon nanobox was designed by Manthiram and co‐workers and it delivered a high initial specific capacity of 1366 mAh g^−1^ at 0.1 C with a retention of 85.3 % after 200 cycles.^[7a]^ Later on, Sun et al. revealed that replacement of Fe_3_O_4_ with Fe_2_N inside the carbon nanobox shell could further improve the cycling stability and rate capability of Li−S batteries, because Fe_2_N exhibited stronger chemical binding and effective catalytic activity for LiPSs.^[9a]^ Besides, Zhang and Tran found that sulfurphilic FeS_2_ particles as additive in the sulfur cathode can improve the long‐term cycling stability of Li−S batteries.^[17]^ Also, Xi et al. prepared the composite of FeS_2_/FeS/S by simple ball milling of Na_2_S, S and FeCl_3_, revealing that FeS_2_ exhibited a stronger adsorption ability than FeS according to their theoretical results with specific electrochemical performance of each sulfide unidentified.^[18]^ Although excellent performance has been achieved, it is difficult to compare and define the optimal composition among various sulfur host materials because of the large variations in their nanostructures. Consequently, it significantly prevents the rational design of electrode materials for Li−S batteries. To address the challenge, systematic research on the chemical composition of host materials with the same nanostructure is required to optimize the metal‐based compounds for Li−S batteries, which, however, remains underdeveloped, especially for Fe‐based compounds. Cui and co‐workers quantitatively compared adsorption ability of a series of metal‐based compounds to LiPSs, revealing that there is an order magnitude of difference between the poor adsorption compounds and strong adsorption ones.^[19]^ Moreover, Qian and co‐workers revealed that among four types of cobalt‐based compounds (CoP, Co_4_N, CoS_2_, and Co_3_O_4_), S@rGO/CoP display best rate capability for Li−S batteries due to moderate adsorption ability and superior diffusion dynamics.^[20]^

**Scheme 1:**
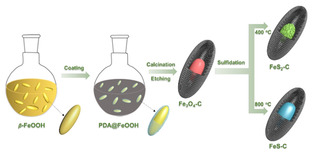
Synthetic routes to yolk‐shell Fe_3_O_4_−C, FeS−C, and FeS_2_−C nanospindles.

In this work, we synthesized three types of yolk‐shell structured iron‐based compounds (Fe_3_O_4_, FeS, and FeS_2_), which were encapsulated into hollow carbon nanospindles, aiming for systematical investigation on their efficiency in suppressing the LiPSs shuttling effect and electrochemical performance in Li−S batteries. The hollow carbon shell provides the physical confinement of LiPSs, while the iron‐based compounds enable the chemical adsorption. The synthesis is scalable and the procedure is depicted in Scheme 1.

Firstly, the colloidal FeOOH nanospindles were synthesized in a large scale with hydrolysis of FeCl_3_ in aqueous solution,^[21]^ which were then coated with a thin polydopamine (PDA) layer as carbon source. Along with calcination under argon and partially etching with HCl, yolk‐shell Fe_3_O_4_−C nanoparticles were obtained. Further calcination of the mixture of sulfur and Fe_3_O_4_−C particles under argon at different temperatures leads to yolk‐shell FeS_2_−C (at 400 °C) and FeS−C (at 800 °C) particles, respectively, without destroying the nanostructure. This synthetic route enables us to directly compare the specific effect of iron oxide and sulfides on the electrochemical performance of Li−S batteries. The influence of material properties (i. e., affinity to LiPSs and conductivity) on the specific capacity, cycling stability, and rate capability has been systematically studied. Our research provides fundamental information for the rational design of efficient sulfur cathode. Besides, the synthesis of yolk‐shell iron‐based composite particles is simple, scalable, and broadly applicable, creating possibilities for mass production of various functional nanomaterials for different applications.

## Results and Discussion

**Figure 1:**
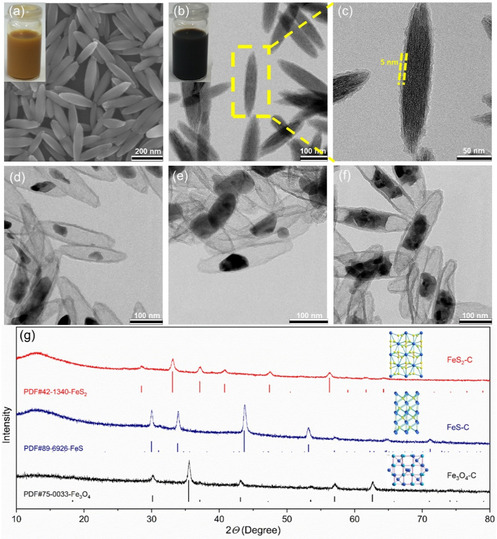
Materials characterization. SEM image of the FeOOH nanospindles (Inset is the photograph of the FeOOH particles dispersed in water) (a); TEM images of the PDA‐coated FeOOH (Inset is the photograph of the PDA‐coated FeOOH particles dispersed in water; b,c), Fe_3_O_4_−C (d), FeS−C (e), and FeS_2_−C nanospindles (f); XRD patterns of these materials (g).

FeOOH nanoparticles were chosen as templates and iron source since they can be easily synthesized in a large scale. Specifically, colloidal β‐FeOOH nanospindles with uniform size and morphology can be obtained by hydrolysis of FeCl_3_ in aqueous solution in the presence of surfactant CTAB under mild condition. The obtained β‐FeOOH nanoparticles are colloidal stable in aqueous solution (Figure 1a, inset). They can be further converted into iron oxide or sulfides through specific post‐treatment. The β‐FeOOH nanoparticles exhibit a nanospindle shape with smooth surface and are monodisperse with a length of about 250 nm and a width of about 50 nm (Figure 1a). The XRD patterns in Figure S1 are indexed to pure beta‐phase FeOOH. In the next step, a thin layer of PDA has been coated on the surface of FeOOH nanospindles using dopamine as monomer in tris buffer solution (pH 8.5). PDA is often used as source for N‐doped porous carbon.^[22]^ Heteroatom doping with nitrogen can increase the adsorption capability of carbon‐based materials to LiPSs due to the increased polarity. In our work, the PDA shell can maintain the nanospindle shape and prevent the aggregation of iron‐based particles during calcination. The color of the FeOOH solution turns from yellow to black (Figure 1b, inset), indicating the successful encapsulation of PDA. The TEM image shown in Figure 1c confirms that a PDA layer of 5 nm has been uniformly coated on the surface of FeOOH nanospindle.

Calcination of PDA@FeOOH under argon at 550 °C leads to the formation of yolk‐shell iron oxide‐carbon nanoparticles. The nanospindle shape of the composite particles remains almost unchanged after calcination, which contains an iron oxide core coated with a 5 nm carbon shell (Figure 1d and Figure S2a). Void spaces in the particles are created due to the structural collapse during the decomposition of FeOOH. The influence of calcination temperature on the formation of iron oxide has been investigated in detail. Iron oxide with a pure Fe_3_O_4_ phase can be obtained in the range of 500–550 °C as demonstrated by the XRD patterns in Figure S3. Additional phases of FeO and Fe appear at 600 °C. Further increase of temperature to 650 °C leads to the complete decomposition of Fe_3_O_4_ with the formation of a mixture of FeO and Fe. In all cases, the final product maintains the yolk‐shell nanostructure. Large voids are created for particles calcined above 600 °C (Figure S2c,d), which may be due to the reduction of iron oxide as shown by XRD. Therefore, the nanoparticles calcined at 550 °C with a pure Fe_3_O_4_ phase were used for further investigation.

To avoid the volume expansion during sulfidation as reported by Liu et al.,^[23]^ the obtained Fe_3_O_4_−C yolk‐shell nanoparticles have been further treated with HCl solution to etch part of Fe_3_O_4_ particles and to create extra void spaces. No obvious extra voids are observed in the particles after etching with 2 M HCl for 1 h (Figure S4). Extra void spaces are created with the core size reduced to 30–50 nm after etching for 2 h. Further increasing the etching time to 3 h leads to the removal of the Fe_3_O_4_ core particles, leaving hollow carbon spindles (Figure S4c). Hence, the optimized etching time was determined as 2 h for further sulfidation process. It is worth noting that the crystal structure of the particles remains the same as the pristine Fe_3_O_4_ after etching process, as shown by XRD (Figure S5).

The yolk‐shell Fe_3_O_4_−C particles have been further sulfidated at 400 and 800 °C to generate FeS_2_−C and FeS−C particles with similar yolk‐shell morphologies, respectively. Elemental sulfur powder has been applied as the sulfur source, which is widely used to synthesize different iron sulfides due to its high reactivity.[^23^, ^24^] For the FeS_2_−C nanoparticles, the mechanism of sulfidation at 400 °C is given by Equation 1:

(1) $$\mathrm{Fe}{}_{3}O{}_{4}+8S\to 3\mathrm{FeS}{}_{2}+2\mathrm{SO}{}_{2}↑$$

With further increasing the calcination temperature, FeS_2_ starts to gradually decompose into FeS and sulfur (550 °C) as shown by Equation 2:

(2) $$\mathrm{FeS}{}_{2}\to \mathrm{FeS}+S↑$$

After sulfidation, the FeS−C and FeS_2_−C particles exhibit a similar morphology as that of Fe_3_O_4_−C nanoparticles, as shown by TEM (Figure 1e,f). From the overview of the SEM images (Figure S6) of the yolk‐shell nanoparticles, no aggregation of iron sulfide is observed due to the protection from the carbon shell. Interestingly, the yolk part of the FeS_2_−C sample is composed of several FeS_2_ nanoparticles with a size of 20–30 nm. This could be caused by the pulverization during the sulfidation process of iron oxide.

**Figure 2:**
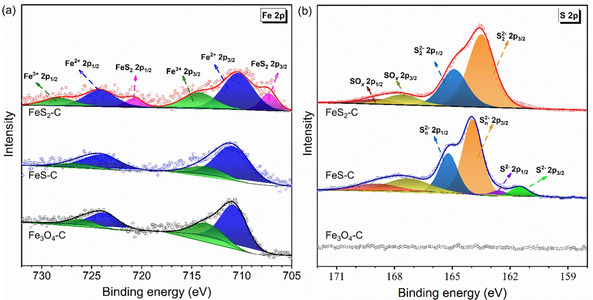
XPS spectra of Fe 2p (a) and S 2p (b) in the Fe_3_O_4_−C, FeS−C, and FeS_2_−C nanoparticles.

The formation of iron sulfides is further confirmed by the XRD patterns (Figure 1g). For the sample sulfidated at 400 °C, the collected patterns are well indexed to pyrite phase FeS_2_ (PDF#42‐1340), indicating the complete conversion of Fe_3_O_4_ into FeS_2_. After sulfidation at 800 °C, troilite phase FeS (PDF#89‐6926) is obtained. The ball‐and‐stick structure models of the crystal structures of magnetite Fe_3_O_4_, troilite FeS, and pyrite FeS_2_ are shown in the inset of Figure 1g. The pyrite FeS_2_ consists of disulfide S_2_^2−^ moieties (S−S, depicted in orange), while the sulfur atoms in troilite FeS are only bonded with iron atoms in the form of S^2−^ instead of S_2_^2−^. Metallic bonding between iron atoms (Fe−Fe, depicted in gray) exists in the crystal structure of FeS, which contributes to a higher electronic conductivity than that of pyrite FeS_2_ and Fe_3_O_4_. To further explore the bonding characteristics, X‐ray photoelectron spectroscopy (XPS) and Raman spectroscopy have been performed, respectively. From the Fe 2p spectra of Fe_3_O_4_−C (Figure 2a), the peaks at 726.1 and 713.8 eV are assigned to Fe 2p_1/2_ and Fe 2p_3/2_ of Fe^3+^ states, respectively, while the peaks at the binding energies of 710.9 eV and 723.8 eV are ascribed to Fe^2+^ states.[^7a^, ^25^] The existence of Fe^3+^ in iron sulfides arises from the surface oxidation of FeS and FeS_2_ nanoparticles. Besides, in the spectra of FeS_2_−C nanoparticles, two additional peaks are observed at 707.4 and 720.7 eV, which are ascribed to the 2p_3/2_ and 2p_1/2_ regions of Fe in pyrite FeS_2_, respectively.^[26]^ In the high‐resolution spectrum of S 2p (Figure 2b), the peaks at 162.6 eV and 161.4 eV correspond to S^2−^ 2p_1/2_ and S^2−^ 2p_3/2_ of FeS, respectively. The detected polysulfide (S_n_^2−^) and sulfate (SO_*x*_) in the FeS−C sample can be caused by the surface oxidation. In the S 2p spectra of FeS_2_−C sample, two characteristic peaks of S_2_^2−^ 2p_1/2_ and S_2_^2−^ 2p_3/2_ are found at 164.9 and 163.5 eV, respectively. In the Raman spectra shown in Figure S7, the peak at 378.1 cm^−1^ in the FeS_2_−C sample is assigned to the A_1g_ of FeS_2_, which is induced by the S−S in‐phase stretching vibration.^[27]^ Two characteristic peaks of FeS are observed at 222 cm^−1^ and 288 cm^−1^ in the Raman spectrum of the FeS−C sample, while the peak at around 372 cm^−1^ belongs to the polysulfide S_n_^2−^.^[28]^ In the spectra of Fe_3_O_4_−C, strong peaks from Fe_2_O_3_ are presented while only weak peaks of Fe_3_O_4_ are found, possibly due to the laser induced conversion of Fe_3_O_4_ into Fe_2_O_3_.^[29]^ Moreover, there are two strong bands at 1345 and 1587 cm^−1^ in all samples, which correspond to the D and G bands of the carbon shell, respectively.

**Figure 3:**
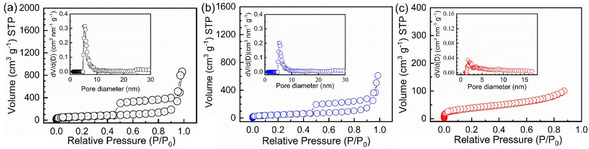
Nitrogen adsorption‐desorption isotherms of Fe_3_O_4_−C (a), FeS−C (b), and FeS_2_−C (c) nanoparticles and corresponding pore size distribution curves as insets.

N_2_ adsorption‐desorption measurements have been conducted to further measure the specific surface area and pore size distribution of these materials (Figure 3). The Brunauer‐Emmett‐Teller (BET) specific surface area of Fe_3_O_4_−C nanoparticles is 223.24 m^2^ g^−1^ and the average pore size is around 5–6 nm. After sulfidation of Fe_3_O_4_−C, the specific surface areas of FeS−C and FeS_2_−C decrease to 162.96 and 134.4 m^2^ g^−1^, respectively. This is due to the increased mass of the core particles after the sulfidation process. The pore size distribution in the FeS_2_−C sample decreases to around 2–3 nm, because the mesopores could be blocked by the FeS_2_ agglomerates.

**Figure 4:**
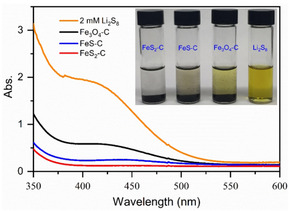
UV/Vis spectroscopy of 2 mM Li_2_S_8_ in DOL/DME (1 : 1 v/v) and the solutions after the addition of Fe_3_O_4_−C, FeS−C, and FeS_2_−C for 2 h with the same surface area of 2 m^2^ based on their BET results. Inset: photographs of the solutions.

Figure 4 shows the photograph of the adsorption of LiPSs onto Fe_3_O_4_−C, FeS−C, and FeS_2_−C. 2 mM Li_2_S_8_ solution (3.5 mL) in DME/DOL was mixed with different host materials. To reveal the affinity of each material to LiPSs, the host materials with the same specific surface area were applied for the adsorption based on the obtained BET specific surface area.^[19]^ After aging for 2 h in the glove box, the color of the supernatant liquid containing FeS_2_−C and FeS−C nanoparticles turns into colorless from yellow, indicating their strong adsorption capability. While the color of the supernatant with Fe_3_O_4_−C nanoparticles slightly bleaches, suggesting its weak affinity to LiPSs. The UV/Vis spectra of these supernatant solutions in Figure 4 confirm that the LiPS concentration in the FeS_2_−C sample is reduced notably.

**Figure 5:**
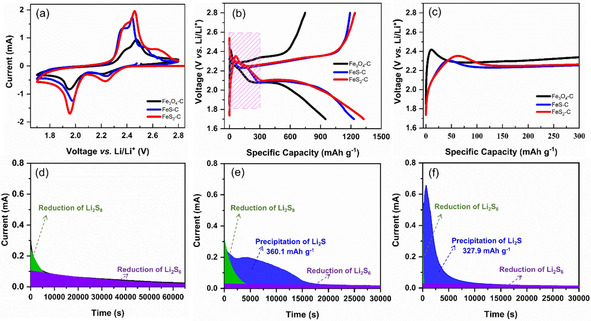
CV curves (a) scanned at 0.1 mV s^−1^, galvanostatic charge/discharge profiles at 0.1 C (b), the enlarged rectangle region (c) of lithium‐sulfur batteries with the S/Fe_3_O_4_−C, S/FeS−C, and S/FeS_2_−C nanospindles as cathode in the range of 1.7–2.8 V vs. Li/Li^+^; The potentiostatic discharge curves of Li_2_S_8_ solution at 2.05 V on different electrodes: Fe_3_O_4_−C (d), FeS−C (e), and FeS_2_−C (f).

To evaluate the effect of different iron compounds on the efficiency of suppressing the LiPSs, and, eventually, on performance of Li−S batteries, as‐prepared composites of S/Fe_3_O_4_−C, S/FeS−C, and S/FeS_2_−C with the same sulfur loading have been used as cathode. The sulfur contents in these different host materials are determined by thermogravimetry analysis (TGA; Figure S8). Figure 5 shows the cyclic voltammetry (CV) curves of the S/Fe_3_O_4_−C, S/FeS−C, and S/FeS_2_−C cathodes at a scan rate of 0.1 mV s^−1^ in the range of 1.7‐2.8 V vs. Li/Li^+^, which are used to investigate the specific electrochemical reactions inside a Li−S battery. All of them exhibit the typical redox peaks originating from the multistep conversion reactions of sulfur into Li_2_S. Specifically, during the initial cathodic scan in the assembled batteries with S/FeS−C and S/FeS_2_−C as cathodes, the two main reduction peaks located at 2.30 and 2.01 V, corresponding to the conversion of sulfur to long‐chain LiPSs and then lower‐order sulfides (Li_2_S_2_ and Li_2_S). In the subsequent anodic scan, the sharp oxidation peak at 2.42 V is due to the transformation of Li_2_S to LiPSs and ultimately to elemental sulfur. In the second cycle, the two cathodic peaks shift to a higher potential value and the anodic peaks shift to a lower electrode potential, indicating a reduced polarization, which is believed to be the re‐accommodation of sulfur after the initial activation cycle.^[30]^ No additional peak from parasitic reactions on the host materials has been observed in the CV profiles. It is worth noting that overlapping of the CV curves in the subsequent cycles, seen in the Figure S9, suggests a good reversibility of the charging‐discharging process. Compared with the iron sulfides, the S/Fe_3_O_4_−C cathode exhibits a much weaker peak at around 1.95 V, indicating the sluggish kinetics of the conversion reaction from long chain LiPSs into Li_2_S.^[9a]^

The galvanostatic charge‐discharge curves of the initial cycle at 0.1 C are shown in Figure 5b. All of them exhibit one typical charge plateau and two discharge plateaus of Li−S batteries. The two stabilized plateaus located at 2.30 and 2.10 V correspond to the transformation of sulfur to long‐chain LiPSs and their conversion to short‐chain lithium sulfides, respectively, which is well consistent with the CV results. The initial specific discharge capacities of the S/Fe_3_O_4_−C, S/FeS−C, and S/FeS_2_−C electrodes at 0.1 C are 948.1, 1229.9, and 1326.4 mAh g^−1^, respectively. Compared to the typical voltage profile of Li−S batteries, the discharge plateau in Figure 5b appears some shorter followed with a sloped tail. This could be fundamentally related to the nanosizing effect of sulfur trapped within the yolk‐shell nanospindles, which increases the surface area of sulfur, resulting in the enhanced surface storage of Li‐ions and suppressed phase transition.^[31]^ Besides, the initial charge curves of the three electrodes inside the magenta rectangular area in Figure 5b are enlarged and plotted in Figure 5c. As it shown, the S/Fe_3_O_4_−C electrode exhibits the highest charging potential barrier to 2.42 V during the initial charging process, indicating the slowest redox kinetics of the conversion reaction from Li_2_S to S.^[32]^ A lower voltage barrier is observed in iron sulfide‐based electrodes due to their higher conductivity than that of Fe_3_O_4_. In addition, the potential difference at half value of the initial charge capacity of the S/FeS−C and S/FeS_2_−C electrodes are 216.3 and 217.6 mV, respectively, which is much lower than that of S/Fe_3_O_4_−C electrode (267.2 mV), indicating a lowered polarization and a facilitated electrochemical redox reaction for the iron sulfide‐based cathodes.

To further elucidate the catalytic effects of the iron‐based nanoparticles, the Li_2_S precipitation and dissolution experiments have been conducted through potentiostatic discharge at 2.05 V and charge at 2.4 V, respectively, according to the previous reports.^[33]^ For the Li_2_S precipitation, the observed peaks in the current‐time profiles (Figure 5d‐f) during the potentiostatic discharging process are indicators for the formation of Li_2_S.^[33a]^ Among them, the FeS_2_−C and FeS−C electrodes show the peaks of Li_2_S precipitation with a precipitation capacity of 327.9 and 360.1 mAh g^−1^, respectively. The absence of the Li_2_S precipitation peak in the Fe_3_O_4_−C electrode indicates no effective Li_2_S precipitation on the cathode side, which could be ascribed to two reasons. One is that a large portion of the LiPSs formed during galvanostatic discharge process might have diffused to the anode side because of the poor adsorption capability of the Fe_3_O_4_−C nanoparticles to LiPSs, leading to the loss of active materials. The other is that the nucleation process of Li_2_S on the Fe_3_O_4_−C electrode is sluggish. Similar results have also been found in the Li_2_S dissolution process (Figure S10a). Interestingly, the Li_2_S precipitation and dissolution peaks in the FeS_2_−C electrode appear much earlier and stronger than that of the FeS−C and Fe_3_O_4_−C electrode, suggesting that FeS_2_ can significantly accelerate the conversion reaction between Li_2_S and LiPSs. Besides, to investigate the effects of the yolk‐shell nanoparticles on the liquid‐liquid transformation of LiPSs, symmetric cells with Li_2_S_6_ electrolyte have been measured using CV scanned at 10 mV s^−1^, as seen in Figure S10b. It is found that iron sulfides exhibit a much higher current density than that of Fe_3_O_4_−C electrode, indicating enhanced redox kinetics between liquid‐phase LiPSs.

**Figure 6:**
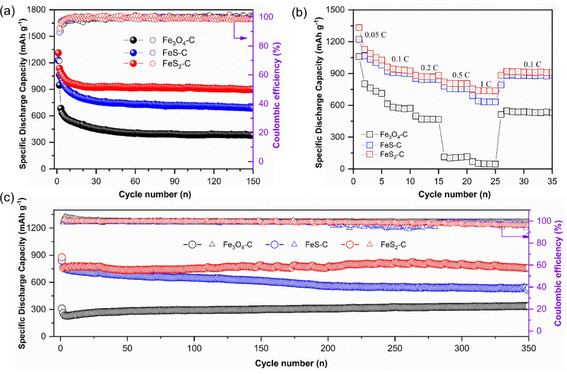
Cycling performance at 0.1 C (a), rate capability (b), and long‐term cycling at 0.5 C (c) of Li−S batteries with the S/Fe_3_O_4_−C, S/FeS−C and S/FeS_2_−C nanospindles, noted in figure, as cathode within the electrochemical potential range of 1.7–2.8 V vs. Li/Li^+^.

The cycling performance and rate capability of these cathodes have been compared as shown in Figure 6a. The battery with S/FeS_2_−C cathode exhibits the highest specific capacity of 930 mAh g^−1^ after 100 charge‐discharge cycles, which is much higher than that of S/Fe_3_O_4_−C (385.8 mAh g^−1^) and S/FeS−C (718.1 mAh g^−1^) cathodes. The excellent cycling stability of S/FeS_2_−C electrode could originate from the better adsorption capability of FeS_2_ to the LiPSs. The rate capability of S/Fe_3_O_4_−C, S/FeS−C, and S/FeS_2_−C are assessed at different discharge rates from 0.1 to 1 C (Figure 6b). Both S/FeS−C and S/FeS_2_−C electrodes show better rate capabilities than S/Fe_3_O_4_−C. Because of the poor confinement ability to LiPSs, the Fe_3_O_4_−C electrode exhibits a fast capacity decay at 0.05 C in the rate capability test, leading to a much lower retained capacity at 0.5 C than that achieved from galvanostatic cycling at the same rate (Figure 6c).

To further interpret this phenomenon, electrochemical impedance spectroscopy (EIS) of Li−S batteries with different host materials have been investigated at open circuit voltage before cycling (Figure S11). The S/Fe_3_O_4_−C cathode has a high polarization and charge transfer resistance (*R*_ct_; as seen from the semicircles in the Nyquist plots) compared to the S/FeS−C and S/FeS_2_−C electrodes, which is responsible for its poor rate capability. The *R*_ct_ of the S/FeS−C cathode is, though slightly lower than, comparable to that of the S/FeS_2_−C cathode. The FeS_2_‐based electrode possesses better rate capability than that of the FeS‐based electrode because the FeS_2_−C nanoparticles have more significant catalytic effect in accelerating the nucleation process of Li_2_S. Besides, the FeS_2_−C nanoparticles show a better adsorption capability to LiPSs than that of FeS−C, leading to less sulfur loss on the cathode side and hence improved capacity.

Furthermore, long‐term cycling performance of batteries with different electrodes at 0.5 C is presented in Figure 6c. Both iron sulfide‐based cathodes achieve much higher specific discharge capacity than that of Fe_3_O_4_. To be specific, the S/FeS_2_−C and S/FeS−C electrodes obtain initial specific discharge capacities of 877.6 mAh g^−1^ and 841.9 mAh g^−1^, respectively, while the S/Fe_3_O_4_−C electrode only delivers an initial specific discharge capacity of 311 mAh g^−1^. The slight capacity increase during cycling at 0.5 C, especially for the Fe_3_O_4_−C/S electrode, is due to the activation process induced by sulfur redistribution.^[34]^ The S/FeS−C electrode undergoes a gradual capacity fading along cycling, while the specific capacity of the S/FeS_2_−C electrode appears rather stable. After 350 cycles, the specific discharge capacity of the S/FeS−C electrode is only 537.8 mAh g^−1^ with a retention ratio of 63.9 %, while the S/FeS_2_−C electrode delivers a specific discharge capacity of 761.2 mAh g^−1^ with a retention ratio of 86.7 %. This demonstrates that a strong adsorption ability of the cathode material to LiPSs could play a crucial role in achieving long‐term cycling stability.

## Conclusion

In summary, three iron oxide/sulfide‐based sulfur host materials with the same yolk‐shell morphology have been synthesized by a simple colloidal route using FeOOH nanoparticles as a template. The same morphology allows for systematic investigation of the effect of chemical composition on the electrochemical performance of Li−S batteries. FeS_*x*_‐based cathodes exhibited a higher specific capacity and better rate capability than Fe_3_O_4_, since they have not only stronger chemical adsorption ability to LiPSs but also higher conductivity. Moreover, we found that the FeS_2_ nanoparticles could significantly accelerate the conversion of LiPSs into Li_2_S. Benefiting from the synergistic chemical adsorption and catalytic effect of FeS_2_ and the physical confinement of the carbon shell to suppress shuttle effects, the S/FeS_2_−C electrode delivered an initial specific discharge capacity of 877.6 mAh g^−1^ at 0.5 C and a retention ratio of 86.7 % after 350 cycles. This work provides fundamental understanding of the specific effect of chemical composition of sulfur host materials beyond nanostructure on the battery performance, which provides an approach for the rational design of efficient cathode materials towards systematic improvement of Li‐S batteries.

## Experimental Section

### Materials

Iron chloride (FeCl_3_, 97 %), cetyltrimethylammonium bromide (CTAB), sublimed sulfur powder, lithium nitrate (LiNO_3_), dopamine hydrochloride, anhydrous ethanol, bis(trifluoromethane)sulfonimide lithium salt (LiTFSI), polyvinylidene fluoride (PVDF), *N*‐methyl‐2‐pyrrolidone (NMP), 1, 2‐dimethoxyethane (DME), and 1, 3‐dioxolane (DOL) were purchased from Sigma‐Aldrich. Hydrochloric acid solution (37 %) was purchased from Alfa‐Aesar. All chemicals were used without any further purification.

### Syntheses

**FeOOH nanospindles**: The synthesis of FeOOH nanospindles was based on the previous report with a minor modification.^[21]^ Specifically, analytical grade iron chloride (3.9 g) was firstly dissolved in distilled water (240 mL), then CTAB powder (6.0 g) was added. The whole mixture was stirred for 30 min at room temperature to form a homogeneous solution. Then, it was heated in an oil bath at 60 °C for 15 h. After it was centrifuged and washed with water for several times, the colloidal solution of FeOOH in water was obtained through ultrasonication.

**PDA‐coated FeOOH nanospindles**: FeOOH nanospindle colloidal aqueous solution (2.4 mL; solid content of FeOOH: 12.6 g L^−1^) was dispersed into a tris‐buffer solution (97.6 mL, pH 8.5–8.6). After ultrasonication for 2 h, 20 mg dopamine hydrochloride was added into the mixture and stirred at room temperature for 15 h with a speed of 450 rpm. Then, the PDA‐coated FeOOH nanospindles were obtained by centrifuging at 7000 rpm for 10 min and followed by washing with water for three times and freeze drying.

**Fe_3_O_4_−C yolk‐shell nanoparticles**: 150 mg of the obtained PDA‐coated FeOOH particles was put in an alumina crucible and calcined at 550 °C for 2 h at a ramping rate of 1 °C min^−1^ under argon. Then, 300 mg of the calcined sample was immersed into the HCl solution (2 M) at room temperature to partially remove the iron oxide particle. After etching for 2 h, the mixture was centrifuged at 8000 rpm for 10 min and washed with deionized water for several times. Then, it was dried at 80 °C under vacuum. The obtained powder was denoted as yolk‐shell Fe_3_O_4_−C nanoparticle.

**FeS_2_−C and FeS−C yolk‐shell nanoparticles**: 100 mg Fe_3_O_4_−C particle was mixed with elemental sulfur (300 mg) in carbon disulfide (3 mL) solution under stirring. After evaporation of the solvent, the mixture was ground in a mortar for 15 min before the sulfidation process. Then, the mixture was calcined in an alumina crucible at 400 °C and 800 °C for 2 h under argon at a ramping rate of 5 °C min^−1^ to get FeS_2_−C and FeS−C nanoparticles, respectively. After calcination, the obtained sample was washed with carbon disulfide and ethanol for several times to remove the residual sulfur. Then, it was dried at room temperature under vacuum.

### Preparation of sulfur composite cathodes

the cathode was prepared by the conventional solid diffusion method. Firstly, the sublimed sulfur powder was mixed with the host materials in a mass ratio of 6 : 4. After grinding for 30 min, the mixture was sealed into a Teflon container under an argon atmosphere. Then, it was heated at 155 °C for 12 h to incorporate sulfur into the yolk‐shell host materials.

### LiPSs adsorption tests

A Li_2_S_8_ solution was prepared by adding the desired amount of S and Li_2_S powder in a molar ratio of 7 : 1 into the solution of DME and DOL (1 : 1 v/v) followed with stirring at 80 °C for 48 h in the glove box. The host material nanoparticles with the same surface area (2 m^2^, based on their BET results) were added to 5 mM Li_2_S_8_ solution (3.5 mL), respectively. After aging for 12 h in the glove box, the supernatant liquid was sealed in cylinder quartz for UV/Vis spectroscopy test.

### Kinetics of Li_2_S precipitation and dissolution on the host materials

Initially, the host materials were mixed with conductive carbon and PVDF with a weight ratio of 8 : 1 : 1 in NMP for forming a mixed slurry. The slurry was coated onto a carbon‐coated Al foil and subsequently dried at 60 °C under vacuum overnight. The areal loading of host materials is around 0.8 mg cm^−2^ and the diameter of the electrode is 14 mm. Finally, the obtained electrodes were assembled into CR2032 coin cells as cathode with Li foil as the counter electrode. 20 μL Li_2_S_8_ (0.25 M) solution with 1.0 M LiTFSI in solvent of DOL and DME (1 : 1 v/v) was applied as catholyte, and 20 μL control electrolyte without Li_2_S_8_ was used as anolyte. For Li_2_S precipitation, the assembled cells were first discharged galvanostatically at 0.1 C to 2.12 V and then discharged potentiostatically at 2.05 V for Li_2_S nucleation and growth. The current vs. time curve of potentiostatic discharge at 2.05 V was fitted as the sum of two exponential functions (*J*=*A* e^*Bt*^; *A* and *B* are constants, *J* is current, and *t* is time), representing the reduction of Li_2_S_8_ in the beginning and Li_2_S_6_ in the last and a peak resulting from the electrodeposition of Li_2_S.^[33a]^ For Li_2_S dissolution, the assembled batteries were first galvanostatically discharged at 0.1 C to 1.7 V, and subsequently galvanostatically discharged at 0.05 C to 1.7 V for complete transformation of liquid LiPSs into solid Li_2_S. Then, the cells were potentiostatically charged at 2.4 V for oxidization of Li_2_S to soluble polysulfides. The potentiostatic discharge/charge processes were recorded with a Biologic VMP3 electrochemical workstation and terminated after 65000 s.

### Kinetic evaluation of polysulfide conversion

Two identical electrodes, the same as the one for Li_2_S precipitation test, were used as the working and the counter electrode, respectively. 40 μL Li_2_S_6_ (0.417 M, or 2.5 M of sulfur) solution with 1.0 M LiTFSI in solvent of DOL and DME (1 : 1 v/v) was used as electrolyte. The CV curves of assembled symmetric battery were measured with a Biologic VMP3 electrochemical workstation at a scan rate of 10 mV s^−1^ between −0.8 and 0.8 V.

### Electrochemical measurements

For the electrode preparation, the sulfur/host material composite, PVDF, and carbon black were mixed in a mass ratio of 8 : 1 : 1 in NMP to make a slurry. After grinding for 30 min, the slurry was coated onto a carbon‐coated Al foil by the doctor blade method. Then, the electrode was dried at 50 °C under vacuum for 12 h. After that, the electrode was cut into circles with a diameter of 14 mm. The areal loading of sulfur was around 1.0–1.1 mg cm^−2^. CR2025 coin cells were assembled with Li foil as anode and a piece of Celgard 2700 membrane as the separator in an Ar filled glove box (UNIlab plus, M. BRAUN) with H_2_O content < 0.5 ppm and O_2_ content < 0.5 ppm. 1 M LiTFSI in DME/DOL (1 : 1 v/v) with 2 wt % of LiNO_3_ was used as the electrolyte. The volume of electrolyte for each cell was 40 μL. Before electrochemical testing, all the cells were aged at room temperature under open circuit potential for 12 h to let the electrolyte wet the electrode. In this work, the current density 1 C equals 1675 mA g^−1^. The specific capacity calculated based on the mass of sulfur. The galvanostatic charging‐discharging was conducted on a Neware battery testing system at room temperature. The CV curves of the assembled batteries were measured with a Biologic VMP3 electrochemical workstation.

### Characterization

The morphology of the obtained samples was investigated by a LEO 1530 field emission SEM and a JEOL‐2100 TEM (JEOL, GmbH, Eching, Germany) at 200 kV. XRD Patterns were collected using a Bruker D8 diffractometer with Cu_Kα_ radiation. N_2_ adsorption‐desorption isotherms were conducted by using Quantachrome Autosorb‐1 systems at 77 K. Specific surface areas were calculated by using the Brunauer‐Emmett‐Teller (BET) method based on a multipoint analysis. The chemical states of the elements in the samples were characterized using X‐ray photoelectron spectroscopy (XPS) with an ESCA‐Lab‐220i‐XL X‐ray Photoelectron Spectrometer (Thermo Fisher Scientific) with Al_Kα_ sources (*hν*=1486.6 eV). The Raman spectra were obtained using a LabRAM HR Evolution Raman spectrometer with a HeNe laser as the excitation line at *λ*=633 nm. Thermogravimetric analysis was carried out on PerkinElmer (TGA 8000) in the temperature range of 30–900 °C at a heating rate of 10 °C min^−1^ under argon. The electrochemical impedance spectroscopy (EIS) was recorded on GAMRY Interface 1000 within a frequency range from 100 kHz to 0.01 Hz.

## Conflict of interest

The authors declare no conflict of interest.

## Supporting information

As a service to our authors and readers, this journal provides supporting information supplied by the authors. Such materials are peer reviewed and may be re‐organized for online delivery, but are not copy‐edited or typeset. Technical support issues arising from supporting information (other than missing files) should be addressed to the authors.

Supplementary

## References

[1a] M. Armand , J. M. Tarascon , Nature 2008, 451, 652–657; 18256660 10.1038/451652a

[1b] Y. Liu , S. Ma , L. Liu , J. Koch , M. Rosebrock , T. Li , F. Bettels , T. He , H. Pfnür , N. C. Bigall , A. Feldhoff , F. Ding , L. Zhang , Adv. Funct. Mater. 2020, 30, 2002462.

[2a] Y. X. Yin , S. Xin , Y. G. Guo , L. J. Wan , Angew. Chem. Int. Ed. 2013, 52, 13186–13200; 10.1002/anie.201304762 24243546

[2b] S. Evers , L. F. Nazar , Acc. Chem. Res. 2013, 46, 1135–1143. 23054430 10.1021/ar3001348

[3a] W. Ren , W. Ma , S. Zhang , B. Tang , Energy Storage Mater. 2019, 23, 707–732;

[3b] A. Manthiram , S. H. Chung , C. Zu , Adv. Mater. 2015, 27, 1980–2006. 25688969 10.1002/adma.201405115

[4] X. Ji , K. T. Lee , L. F. Nazar , Nat. Mater. 2009, 8, 500–506. 19448613 10.1038/nmat2460

[5] A. Fu , C. Wang , F. Pei , J. Cui , X. Fang , N. Zheng , Small 2019, 15, 1804786. 10.1002/smll.201804786 30721557

[6] J. Zhang , Z. Li , Y. Chen , S. Gao , X. W. D. Lou , Angew. Chem. Int. Ed. 2018, 57, 10944–10948; 10.1002/anie.201805972 29949224

[7a] J. He , L. Luo , Y. Chen , A. Manthiram , Adv. Mater. 2017, 29, 1702707; 10.1002/adma.201702707 28692775

[7b] P. Wang , R. Zeng , L. You , H. Tang , J. Zhong , S. Wang , T. Yang , J. Liu , ACS Nano 2020, 3, 1382–1390;

[7c] C. Zheng , S. Niu , W. Lv , G. Zhou , J. Li , S. Fan , Y. Deng , Z. Pan , B. Li , F. Kang , Q.-H. Yang , Nano Energy 2017, 33, 306–312;

[7d] S. Mei , C. J. Jafta , I. Lauermann , Q. Ran , M. Kärgell , M. Ballauff , Y. Lu , Adv. Funct. Mater. 2017, 27, 1701176;

[7e] J. Zhang , Y. Shi , Y. Ding , W. Zhang , G. Yu , Nano Lett. 2016, 16, 7276–7281; 27736079 10.1021/acs.nanolett.6b03849

[7f] C. Wang , Y. Yi , H. Li , P. Wu , M. Li , W. Jiang , Z. Chen , H. Li , W. Zhu , S. Dai , Nano Energy 2020, 67, 104253.

[8a] B. Guo , S. Bandaru , C. Dai , H. Chen , Y. Zhang , Q. Xu , S. Bao , M. Chen , M. Xu , ACS Appl. Mater. Interfaces 2018, 10, 43707–43715; 30480423 10.1021/acsami.8b16948

[8b] X. Liu , J. Q. Huang , Q. Zhang , L. Mai , Adv. Mater. 2017, 29, 1601759; 10.1002/adma.201601759 28160327

[8c] Z. Liu , X. Zheng , S. Luo , S. Xu , N. Yuan , J. Ding , J. Mater. Chem. A 2016, 4, 13395–13399;

[8d] Z. Xiao , Z. Yang , L. Zhang , H. Pan , R. Wang , ACS Nano 2017, 11, 8488–8498. 28745863 10.1021/acsnano.7b04442

[9a] W. Sun , C. Liu , Y. Li , S. Luo , S. Liu , X. Hong , K. Xie , Y. Liu , X. Tan , C. Zheng , ACS Nano 2019, 13, 12137–12147; 31593436 10.1021/acsnano.9b06629

[9b] C. Ye , Y. Jiao , H. Jin , A. D. Slattery , K. Davey , H. Wang , S. Z. Qiao , Angew. Chem. Int. Ed. 2018, 57, 16703–16707; 10.1002/anie.201810579 30325094

[9c] Z. Li , Q. He , X. Xu , Y. Zhao , X. Liu , C. Zhou , D. Ai , L. Xia , L. Mai , Adv. Mater. 2018, 30, 1804089; 10.1002/adma.201804089 30259567

[9d] X. Yang , S. Chen , W. Gong , X. Meng , J. Ma , J. Zhang , L. Zheng , H. D. Abruña , J. Geng , Small 2020, 16, 2004950. 10.1002/smll.202004950 33155429

[10a] H. Wei , E. F. Rodriguez , A. S. Best , A. F. Hollenkamp , D. Chen , R. A. Caruso , ACS Appl. Mater. Interfaces 2019, 11, 13194–13204; 30912440 10.1021/acsami.8b21627

[10b] F. Zhou , Z. Li , X. Luo , T. Wu , B. Jiang , L. L. Lu , H. B. Yao , M. Antonietti , S. H. Yu , Nano Lett. 2018, 18, 1035–1043; 29300493 10.1021/acs.nanolett.7b04505

[10c] Z. Xiao , Z. Li , P. Li , X. Meng , R. Wang , ACS Nano 2019, 13, 3608–3617. 30864777 10.1021/acsnano.9b00177

[11a] Y. Chen , W. Zhang , D. Zhou , H. Tian , D. Su , C. Wang , D. Stockdale , F. Kang , B. Li , G. Wang , ACS Nano 2019, 13, 4731–4741; 30924635 10.1021/acsnano.9b01079

[11b] S. Huang , Y. V. Lim , X. Zhang , Y. Wang , Y. Zheng , D. Kong , M. Ding , S. A. Yang , H. Y. Yang , Nano Energy 2018, 51, 340–348.

[12a] G. Li , J. Sun , W. Hou , S. Jiang , Y. Huang , J. Geng , Nat. Commun. 2016, 7, 10601; 26830732 10.1038/ncomms10601 PMC4740444

[12b] S. Qi , J. Sun , J. Ma , Y. Sun , K. Goossens , H. Li , P. Jia , X. Fan , C. W. Bielawski , J. Geng , Nanotechnology 2018, 30, 024001; 30378565 10.1088/1361-6528/aae6e5

[12c] Y. Sun , J. Ma , X. Yang , L. Wen , W. Zhou , J. Geng , J. Mater. Chem. A 2020, 8, 62–68.

[13] Y. Zhang , G. Li , J. Wang , G. Cui , X. Wei , L. Shui , K. Kempa , G. Zhou , X. Wang , Z. Chen , Nat. Commun. 2020, 30, 2001165.

[14] J. Shen , X. Xu , J. Liu , Z. Liu , F. Li , R. Hu , J. Liu , X. Hou , Y. Feng , Y. Yu , M. Zhu , ACS Nano 2019, 13, 8986–8996. 31356051 10.1021/acsnano.9b02903

[15] W. Li , Z. Gong , X. Yan , D. Wang , J. Liu , X. Guo , Z. Zhang , G. Li , J. Mater. Chem. A 2020, 8, 433–442.

[16a] Y. Zhang , R. Gu , S. Zheng , K. Liao , P. Shi , J. Fan , Q. Xu , Y. Min , J. Mater. Chem. A 2019, 7, 21747–21758;

[16b] W. Li , Z. Chen , D. Wang , Z. Gong , C. Mao , J. Liu , H. Peng , Z. Zhang , G. Li , J. Power Sources 2019, 435, 226778;

[16c] Y. Boyjoo , H. Shi , E. Olsson , Q. Cai , Z. S. Wu , J. Liu , G. Q. Lu , Adv. Energy Mater. 2020, 10, 2000651;

[16d] P. Jiang , S. Chen , C. Wang , D. Wang , J. Diao , Z. Cao , Z. Lin , Q. Luo , J. Lu , H. Huang , C. Zong , L. Hu , Q. Chen , Mater. Today Sustain. 2020, 9, 100039.

[17] S. S. Zhang , D. T. Tran , J. Mater. Chem. A 2016, 4, 4371–4374.

[18] K. Xi , D. He , C. Harris , Y. Wang , C. Lai , H. Li , P. R. Coxon , S. Ding , C. Wang , R. V. Kumar , Adv. Sci. 2019, 6, 1800815. 10.1002/advs.201800815 PMC6425436 30937253

[19] D. S. Wu , F. Shi , G. Zhou , C. Zu , C. Liu , K. Liu , Y. Liu , J. Wang , Y. Peng , Y. Cui , Energy Storage Mater. 2018, 13, 241–246.

[20] J. Zhou , X. Liu , L. Zhu , J. Zhou , Y. Guan , L. Chen , S. Niu , J. Cai , D. Sun , Y. Zhu , J. Du , G. Wang , Y. Qian , Joule 2018, 2, 2681–2693.

[21] X. Wang , X. Chen , L. Gao , H. Zheng , M. Ji , C. Tang , T. Shen , Z. Zhang , J. Mater. Chem. 2004, 14, 905–907.

[22] K. Qu , Y. Wang , A. Vasileff , Y. Jiao , H. Chen , Y. Zheng , J. Mater. Chem. A 2018, 6, 21827–21846.

[23] Z. Liu , T. Lu , T. Song , X.-Y. Yu , X. W. Lou , U. Paik , Energy Environ. Sci. 2017, 10, 1576–1580.

[24a] Q. Wang , W. Zhang , C. Guo , Y. Liu , C. Wang , Z. Guo , Adv. Funct. Mater. 2017, 27, 1703390;

[24b] Y. Zhao , J. Zhu , S. J. H. Ong , Q. Yao , X. Shi , K. Hou , Z. J. Xu , L. Guan , Adv. Energy Mater. 2018, 8, 1802565.

[25] X. Hu , Y. Liu , J. Chen , J. Jia , H. Zhan , Z. Wen , J. Mater. Chem. A 2019, 7, 1138–1148.

[26] Y. Xi , X. Ye , S. Duan , T. Li , J. Zhang , L. Jia , J. Yang , J. Wang , H. Liu , Q. Xiao , J. Mater. Chem. A 2020, 8, 14769–14777.

[27] L. Wu , N. Y. Dzade , L. Gao , D. O. Scanlon , Z. Öztürk , N. Hollingsworth , B. M. Weckhuysen , E. J. Hensen , N. H. De Leeuw , J. P. Hofmann , Adv. Mater. 2016, 28, 9602–9607. 27628579 10.1002/adma.201602222

[28] A. Matamoros-Veloza , O. Cespedes , B. R. Johnson , T. M. Stawski , U. Terranova , N. H. de Leeuw , L. G. Benning , Nat. Commun. 2018, 9, 3125. 30087338 10.1038/s41467-018-05493-x PMC6081449

[29] Z. X. Yang , K. Qian , J. Lv , W. H. Yan , J. H. Liu , J. W. Ai , Y. X. Zhang , T. L. Guo , X. T. Zhou , S. Xu , Sci. Rep. 2016, 6, 27957. 27296103 10.1038/srep27957 PMC4906393

[30a] Y. Wang , D. Adekoya , J. Sun , T. Tang , H. Qiu , L. Xu , S. Zhang , Y. Hou , Adv. Funct. Mater. 2018, 29, 1807485;

[30b] Y. S. Su , A. Manthiram , Nat. Commun. 2012, 3, 1166. 23132016 10.1038/ncomms2163

[31a] H. Chen , C. Wang , W. Dong , W. Lu , Z. Du , L. Chen , Nano Lett. 2015, 15, 798–802; 25546227 10.1021/nl504963e

[31b] M. Wagemaker , F. M. Mulder , Acc. Chem. Res. 2013, 46, 1206–1215. 22324286 10.1021/ar2001793

[32] G. Zhou , H. Tian , Y. Jin , X. Tao , B. Liu , R. Zhang , Z. W. Seh , D. Zhuo , Y. Liu , J. Sun , J. Zhao , C. Zu , D. S. Wu , Q. Zhang , Y. Cui , Proc. Natl. Acad. Sci. USA 2017, 114, 840–845. 28096362 10.1073/pnas.1615837114 PMC5293031

[33a] F. Y. Fan , W. C. Carter , Y. M. Chiang , Adv. Mater. 2015, 27, 5203–5209; 26257297 10.1002/adma.201501559

[33b] D. Zhang , S. Wang , R. Hu , J. Gu , Y. Cui , B. Li , W. Chen , C. Liu , J. Shang , S. Yang , Adv. Funct. Mater. 2020, 30, 2002471.

[34a] H. Yuan , X. Chen , G. Zhou , W. Zhang , J. Luo , H. Huang , Y. Gan , C. Liang , Y. Xia , J. Zhang , J. Wang , X. Tao , ACS Energy Lett. 2017, 2, 1711–1719;

[34b] M. Zhao , H. J. Peng , Z. W. Zhang , B. Q. Li , X. Chen , J. Xie , X. Chen , J. Y. Wei , Q. Zhang , J. Q. Huang , Angew. Chem. Int. Ed. 2019, 58, 3779–3783; 10.1002/anie.201812062 30548388

