# Supplementary material for: Efficient Sulfur Host Based on Yolk‐Shell Iron Oxide/Sulfide‐Carbon Nanospindles for Lithium‐Sulfur Batteries
Source: ChemSusChem. 2021 Feb 2;14(5):1404–13. doi: 10.1002/cssc.202002731 (PMC7986775; doi:10.1002/cssc.202002731)
Supplement: Supplementary file 1 — Supplementary [file CSSC-14-1404-s001.pdf]

ChemSusChem

Supporting Information

Efficient Sulfur Host Based on Yolk-Shell Iron Oxide/Sulfide-Carbon Nanospindles for Lithium-Sulfur Batteries

Dongjiu Xie, Shilin Mei, Yaolin Xu, Ting Quan, Eneli Härk, Zdravko Kochovski, and Yan Lu*© 2021 The Authors. ChemSusChem published by Wiley-VCH GmbH. This is an open access article under the terms of the Creative Commons Attribution License, which permits use, distribution and reproduction in any medium, provided the original work is properly cited.

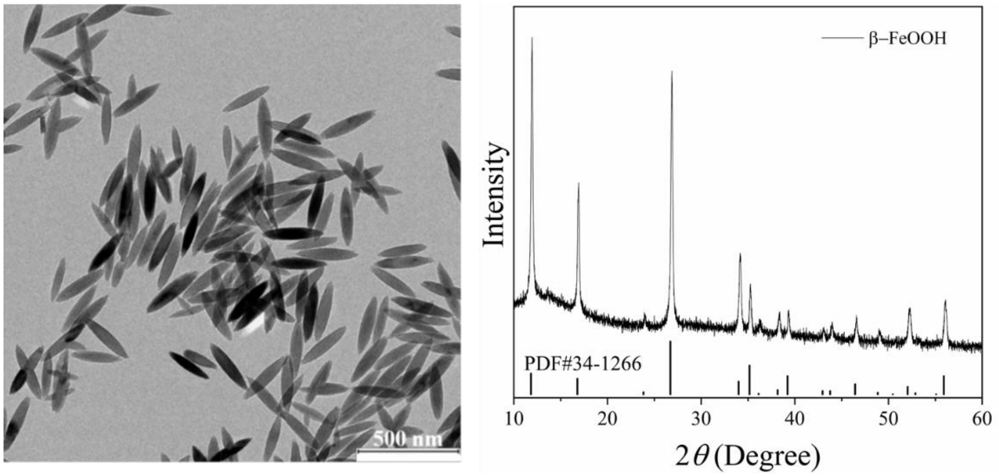

Figure S1. TEM image and XRD patterns of the colloidal *β*-FeOOH nanospindles.

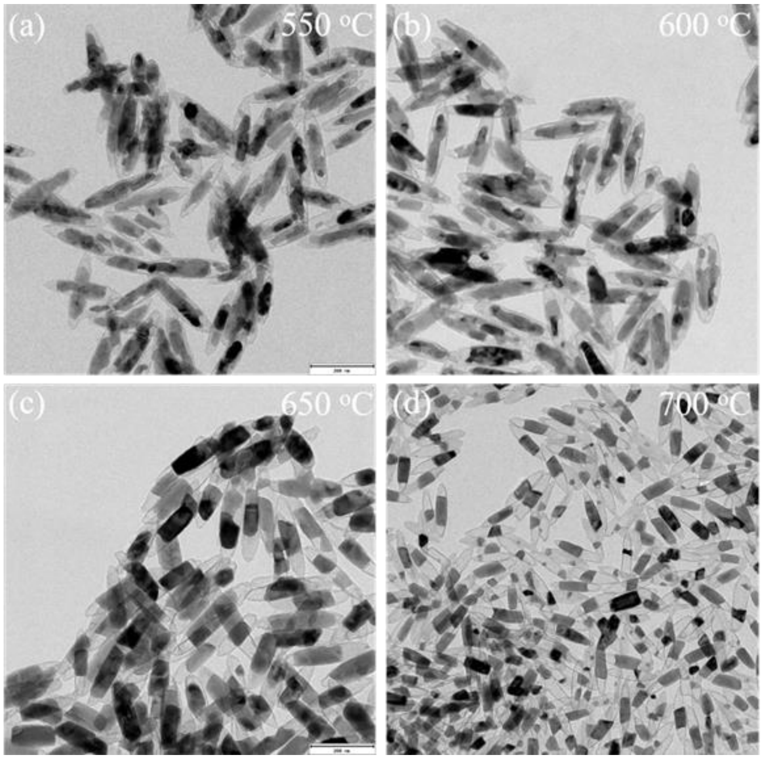

Figure S2. TEM images of the PDA-coated FeOOH after calcination at different temperatures for 2 h with a ramping rate of 1 °C/min under argon.

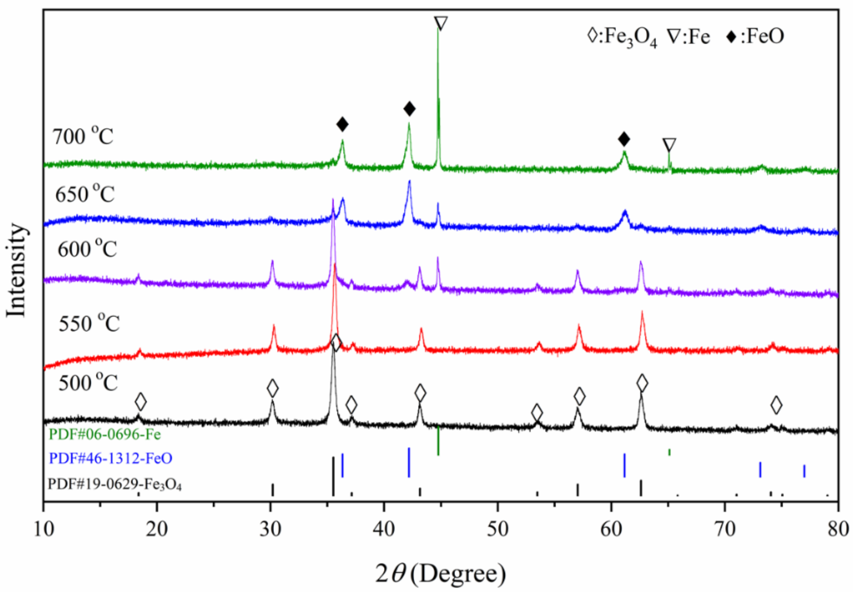

Figure S3. XRD patterns of the PDA-coated FeOOH after calcination at different temperatures for 2 h with a ramping rate of 1 °C/min under argon.

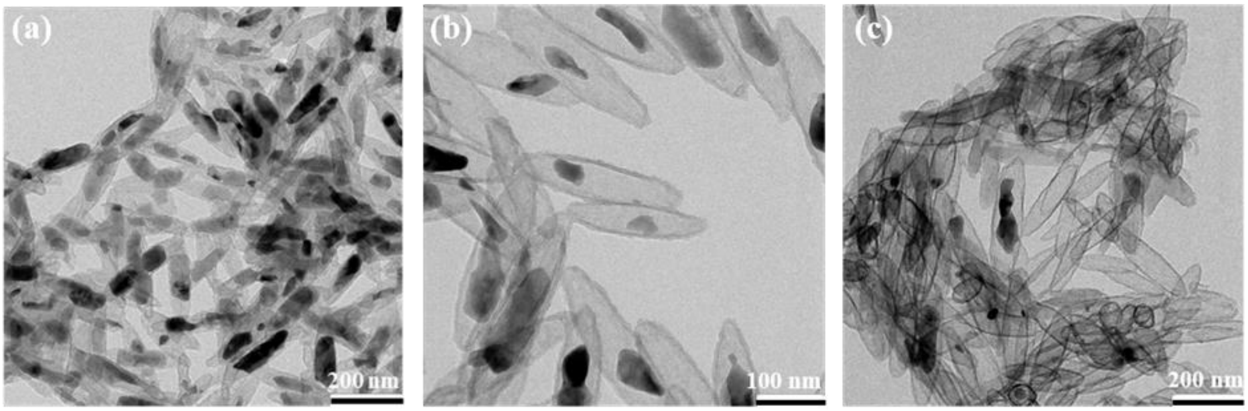

Figure S4. TEM images of the Fe_3O_4-C yolk-shell nanospindles after etching with HCl solution for different durations (1, 2 and 3 h) under a mild mechanical stirring.

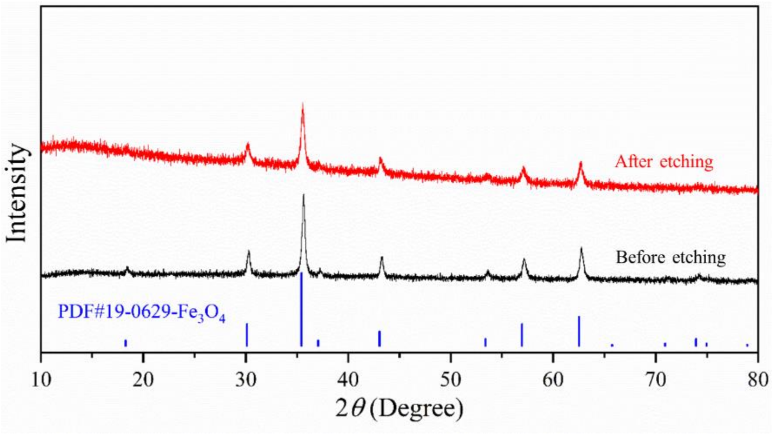

Figure S5. XRD patterns of the Fe_3O_4-C yolk-shell nanospindles before and after etching with HCl solution for 2 h under a mild mechanical stirring.

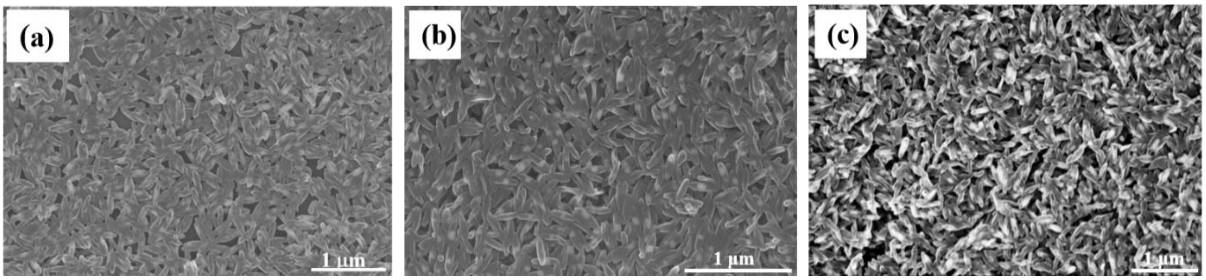

Figure S6. SEM images of the Fe_3O_4-C (a), FeS-C (b), and FeS_2-C (c) nanospindles.

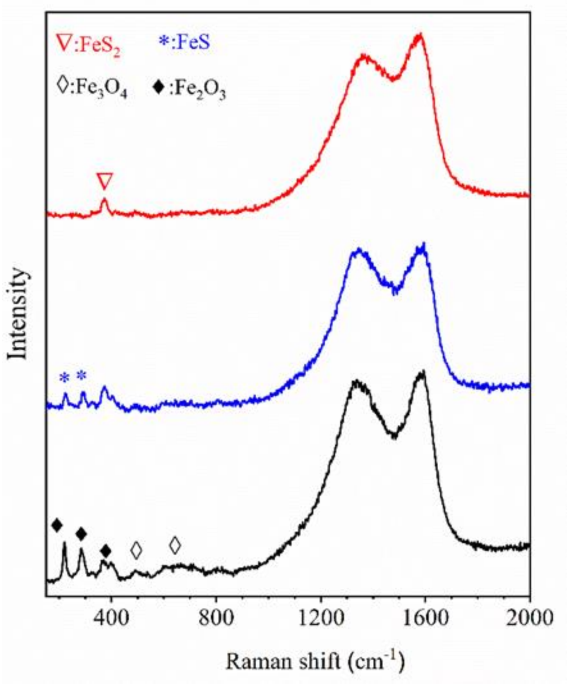

Figure S7. Raman spectra of the Fe_3O_4-C (black line), FeS-C (blue line), and FeS_2-C (red line) nanospindles.

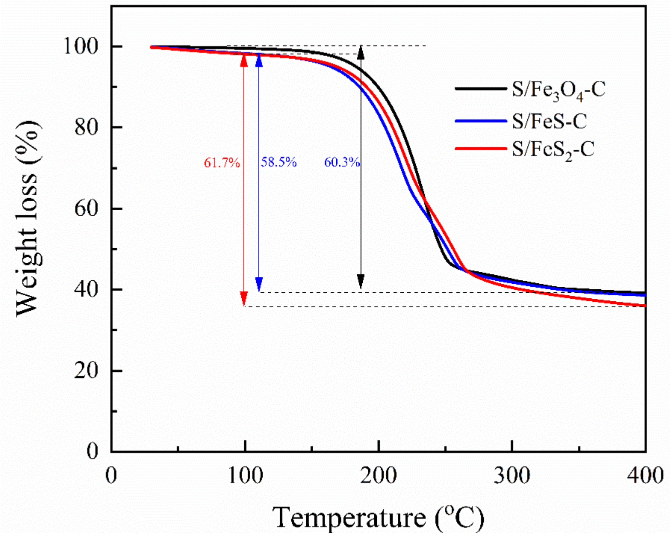

Figure S8. Thermogravimetry analysis curves of the S/Fe_3O_4-C, S/FeS-C, and S/FeS_2-C composites under argon.

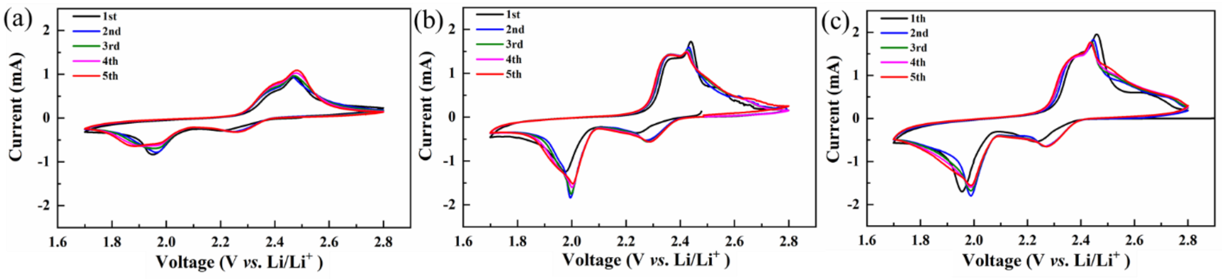

Figure S9. CV curves of the Li-S coin cells with S/Fe_3O_4-C (a), S/FeS-C (b), and S/FeS_2-C (c) composites as cathodes scanned at 0.1 mV s^{-1} in the range of 1.7 - 2.8 V *vs* Li/Li^+.

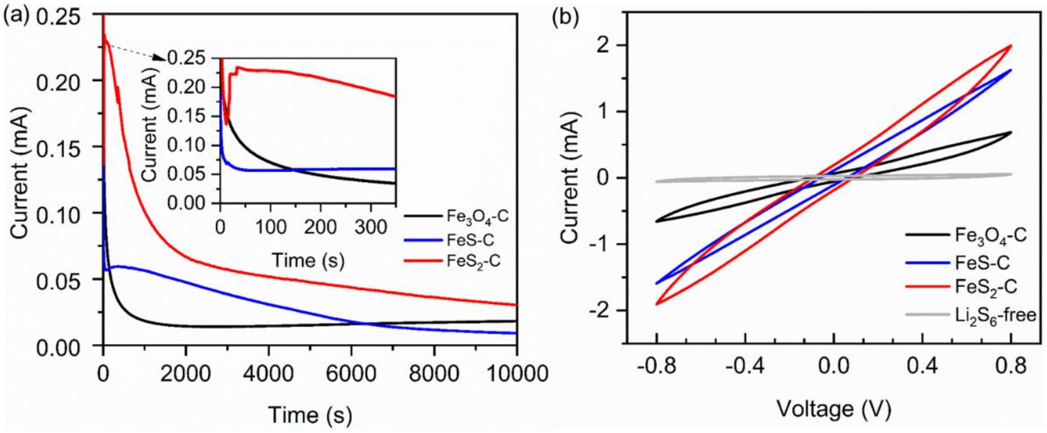

Figure S10. Potentiostatic charge curves (a) of Li_2S_8 solution at 2.4 V *vs*. Li/Li^+ on different electrodes; CV curves (b) of symmetric cells with Li_2S_6 solution as electrolyte scanned at 10 mV s^{-1} between -0.8 and 0.8 V.

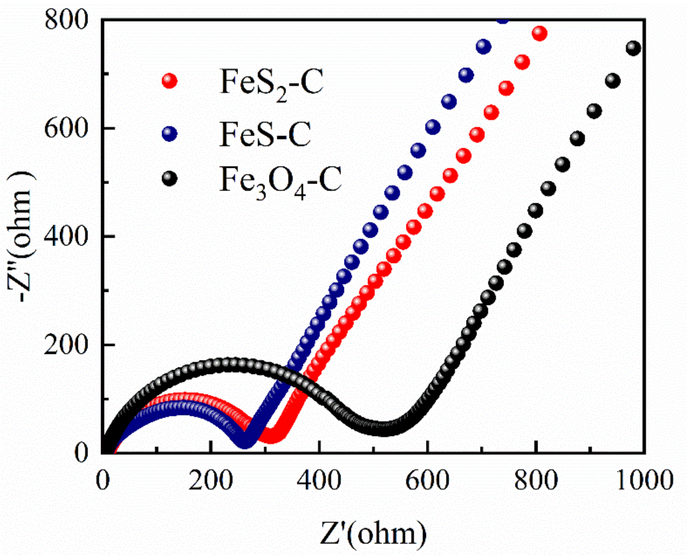

Figure S11. Electrochemical impedance spectroscopy of lithium-sulfur batteries with the S/Fe_3O_4-C, S/FeS-C and S/FeS_2-C nanospindles as cathode before cycling.
